# Measurement of unmet healthcare needs to assess progress on universal health coverage - exploring a novel approach based on household surveys

**DOI:** 10.1186/s12913-023-09542-0

**Published:** 2023-05-23

**Authors:** Alok Ranjan, Sundararaman Thiagarajan, Samir Garg

**Affiliations:** 1 Department of Humanities and Social Sciences, Indian Institute of Technology, Jodhpur, India; 2 State Health Resource Center, Chhattisgarh, India

**Keywords:** Unmet healthcare needs, Universal Health Coverage (UHC), Access to healthcare

## Abstract

**Background:**

Universal Health Coverage (UHC) aims to ensure universal access to quality healthcare according to health needs. The extent to which population health needs are met should be a key measure for progress on UHC. The indicators in use for measuring access mostly relate to physical accessibility or insurance coverage. Or, utilization of services is taken as indirect measure for access but it is assessed against only the perceived healthcare needs. The unperceived needs do not get taken into account. The present study was aimed at demonstrating an approach for measuring the unmet healthcare needs using household survey data as an additional measure of UHC.

**Methods:**

A household survey was conducted in Chhattisgarh state of India, covering a multi-stage sample of 3153 individuals. Healthcare need was measured in terms of perceived needs which would be self-reported and unperceived needs where clinical measurement supplemented the interview response. Estimation of unperceived healthcare needs was limited to three tracer conditions- hypertension, diabetes and depression. Multivariate analysis was conducted to find the determinants of the various measures of the perceived and unperceived needs.

**Results:**

Of the surveyed individuals, 10.47% reported perceived healthcare needs for acute ailments in the last 15 days. 10.62% individuals self-reported suffering from chronic conditions. 12.75% of those with acute ailment and 18.40% with chronic ailments received no treatment, while 27.83% and 9.07% respectively received treatment from unqualified providers. On an average, patients with chronic ailments received only half the medication doses required annually. The latent need was very high for chronic ailments. 47.42% of individuals above 30 years age never had blood pressure measured. 95% of those identified with likelihood of depression had not sought any healthcare and they did not know they could be suffering from depression.

**Conclusion:**

To assess progress on UHC more meaningfully, better methods are needed to measure unmet healthcare needs, taking into account both the perceived and unperceived needs, as well as incomplete care and inappropriate care. Appropriately designed household surveys offer a significant potential to allow its periodic measurement. Their limitations in measuring the ‘inappropriate care’ may necessitate supplementation with qualitative methods.

**Supplementary Information:**

The online version contains supplementary material available at 10.1186/s12913-023-09542-0.

## Background

Ensuring that all those in need of healthcare are able to access quality healthcare according to the need and without financial hardship is the central aim of universal health coverage (UHC) [1, 2]. Hence, the proportion of the population in need of health care which is able to access effective or quality healthcare, should be key measure for assessing progress on UHC. Knowing unmet needs and their magnitude can be useful in deciding policies and planning for the required services.

There are many challenges with measuring healthcare needs [3]. Whereas it is relatively straightforward to measure the numbers who have accessed specific services, it becomes difficult to measure the total health care need or those who had health needs but were *unable* to access appropriate services. In the Indian context, the main source of data on morbidity and utilisation of healthcare is the National Sample Survey (NSS) [4]. This household survey is conducted periodically by India’s central ministry of statistics. Most estimates of the unmet healthcare needs have been calculated based on self-reporting of morbidities. Existing research has shown that the NSS tends to under-report morbidity [5, 6]. Further, self-reporting does not take into account the unperceived healthcare needs.

The conceptualization of access has implications for measuring progress towards UHC [7]. Access has often been defined as the ability to obtain the required services [8]. One type of conceptualisation of access is from a purely supply-side perspective. It deals with the *availability* of services. It relates access to a measure of location [9]. The use of provider to population ratio is also a part of this approach [10]. Another approach is to study the demand side or utilization of services. It could also involve a study of differentials in utilization, especially when equity in access is the focus [11]. Another important determinant of access is *affordability*. Ability to pay for services, supplementing income for those who cannot pay, insurance coverage, eligibility criteria for the publicly funded program or other matters related to cost of care at the time of receiving services come under this [12]. In the utilization of services, there is also the dimension of the fit between people’s healthcare needs and supply of healthcare to them. It conceptualizes access as not merely the availability of services but the quality and information dimensions, which could be termed the *acceptability* of services [13, 14].

It is acknowledged that access is not directly measurable [8]. Hence indirect indicators are used to reflect access [8]. In many situations, effective access is equated with overall utilization rate, which is true for services like immunization and family planning [15]. It is not true for conditions where inappropriate or incomplete care are prevalent or where because of latent needs the numbers needing care are not known. Neither physical access nor insurance coverage nor even utilization rates for different services can be equated with effective access. In the UHC literature, access is encompassed under the term ‘coverage’ [1, 2]. UHC defines three dimensions of ‘coverage’ – what proportion of population is covered, what services are included and the extent to which financial protection is ensured [1, 2]. However, the term ‘coverage’ carries the danger of getting equated with ‘insurance coverage’ i.e., inclusion in health insurance programmes. We term what we try to measure here as ‘service coverage’ or ‘effective access’ and define it as the proportion of individuals in need of healthcare who could utilize appropriate services of sufficient quality [16, 17].

As argued above, the existing approaches to measure the healthcare needs and coverage have serious limitations. Methods need to be developed to capture the unmet needs better. In this study we are proposing that with certain changes in the design of household surveys, we would be able to periodically measure most unmet needs reliably in the Indian context.

Our approach categorizes all healthcare needs into perceived and unperceived needs. Unperceived or latent needs are those health needs where on basic of current medical knowledge healthcare is indicated, but the need has not been recognised either due to lack of screening for it, or inadequate health-seeking due to lack of knowledge or other barriers. These are all unmet needs. Perceived needs can be met (fulfilled) or unmet. Unmet needs could take the form of a) no treatment b) treatment that is incomplete, c) treatment from an unqualified provider and d) treatment that is inappropriate viz. it does not qualify to be called or categorized as “health care that is effective”. In “*incomplete care*” as measured in this study, the individual was unable to continue medication for the prescribed period. Unqualified or informal providers are not a part of the government’s UHC strategy of provision of essential healthcare services, and therefore irrespective of whether their treatment worked, they were for the purpose of this study categorised as part of the gap in services. Formal qualification or training of the provider is also a proxy indicator for measuring health care that is effective. Under “*inappropriate care”*, the individual got the treatment, but it was not in accordance with what is normatively considered as appropriate. We chose to leave out the affordability dimension in this article as sufficient literature is available on out of pocket expenditure in Indian health system [17–19].

## Methods

### Study setting

This study was conducted in Korba district in Chhattisgarh state of India. The district had a population of 1.206 million [20]. It had a significant proportion of the vulnerable communities belonging to the scheduled tribes (40.9%) and the scheduled castes (10.33%) [20]. The roll out of strategies that constitute the Indian road map towards UHC were in a relatively advanced stage in this district. Every resident was officially covered by the government-funded health insurance that covered almost the entire range of secondary and tertiary care services by involving a number of public and private hospitals. There is also a public sector health system with three tiers to provide the primary, secondary and tertiary healthcare. It had the normative density of public-sector facilities including a district hospital that was quality-certified [21, 22].

### Study design and sampling

It was a cross-sectional study. It covered a representative sample of the district. A key purpose of the study was to measure access for the morbidities reported or identified during the survey. At 90% confidence level, we calculated a requirement of around 228 persons with healthcare needs. A total sample requirement of 3000 individuals was estimated to yield the above number. Assuming the average household size as five, 600 households were selected through multistage random sampling.

Selection of villages and urban area was done through proportionate to population size sampling. In the first stage, thirteen rural units (villages) and seven urban units (wards) were selected through systematic random sampling respectively out of the lists of villages and urban-wards of the district. All households in the village/ward were listed, and from these, the required number of 30 households were selected by systematic random sampling. The sampling method was the same as in India’s large morbidity surveys [23]. The survey was able to cover a sample of 3153 individuals belonging to 598 households. Out of the total sample, 2012 individuals were from rural areas, whereas 1141 individuals were from urban areas. The sample thus was 64% rural and 36% urban households, which was designed to be consistent with the proportion reported for the district in the national census [20].

### Data collection

Data was collected using a structured quantitative tool. The interview schedule included demographic details of the households, hospitalization episodes in the last 365 days, acute illness and outpatient visits in the last 15 days, chronic illness, choice of provider, medical and non-medical expenditure and health insurance coverage. The data collection took place between June to October 2018.

The background characteristics of each household were recorded and this included age, sex, residence, caste, education levels, occupation, and stated income (Table 1). In addition, the household’s usual annual per capita consumption expenditure was calculated, and this was used to categorize them into economic quintiles.

As a single question, individuals were asked if they were suffering from any acute illness in the last 15 days. And then they were asked what the disease was? Whatever was stated- symptoms or disease was noted. The response was later categorized into 78 different ailment categories by the principal investigator in consultation with two medical experts.

For each individual, it was asked if they were suffering from a chronic disease. A chronic condition was defined as any illness with duration more than three months. Whether the individual was suffering from a chronic condition was first asked as a single question. It was followed by use of the ‘cue’ or ‘prompt’ to aid recall for those who did not report a chronic ailment in response to the single question. It involved further asking the respondents whether any individual in the family was suffering from any of the following ten chronic diseases or conditions: (1) Hypertension, (2) Diabetes, (3) Kidney or urinary disease, (4) Lung diseases (asthma, chronic obstructive pulmonary diseases and chronic bronchitis), (5) Heart diseases, (6) Stroke, (7) Arthritis or rheumatic conditions, (8) Neurological conditions, (9) Mental illness and (10) Cancer. This is consistent with the methodology followed in Longitudinal Aging Study in India (LASI) and the WHO study of Global Aging and Adult Health (SAGE), two studies that also had a focus on chronic diseases [24]. The aim was also to calculate recall for these ten major chronic conditions compared to the one-line question response. The latter method is used in the NSSO morbidity surveys [4].

Medical management of chronic diseases usually requires taking medication regularly for long periods. For each individual reporting a chronic ailment, the completeness of treatment was taken as the part of the year for which medicine was taken. Data was collected on the access to free medicines and the distance of the closest medicine store.

Hospitalisation, i.e., the utilisation of inpatient care was asked for one year from the survey. However, measuring the need for hospitalisation was found to be difficult. Household respondents found it difficult to specify whether they needed inpatient care or ambulatory care. The type of care (inpatient/outpatient) was mostly decided by the healthcare providers and not the patients.

The choice of service providers during hospitalization and outpatient care was also recorded along with broader public and private categories. Its purpose was to find out the utilisation of healthcare from unqualified providers. According to the existing laws in the state, any medical-practice by a private provider without a government-approved medical degree and registration as a clinical establishment is illegal. There was a likelihood that the household respondents may not be aware whether the private provider they went to was qualified or not. None of the existing national morbidity surveys in India are able to capture this aspect adequately. In order to identify the type of private provider (qualified/unqualified), the names and addresses of the private providers used were asked from the household respondents. Then the government health workers in the concerned areas were asked to confirm whether the private provider was qualified or unqualified.

All household members who were 30 years or above and gave informed consent for participation were screened for hypertension and diabetes. In case of non-availability of the household member, they were followed up three times- once on the same day evening and twice on the following day. Blood pressure equal to or more than 140 (systolic)/90 (diastolic) persistent on repeating measurement thrice with time intervals in case of high blood pressure recording, and random blood glucose of 200 mg/dl or above, were cut-off points for suspecting hypertension and diabetes [25]. Standard protocols as used in earlier National Family Health Surveys (NFHS) were followed for blood pressure and blood glucose measurements[23]. If the measurement indicated presence of the disease, the participant was referred to the nearest government health facility and the local community health worker was informed to facilitate follow-up care.

The standard “Patient Health Questionnaire-9 (PHQ-9)” was used to screen for depression [26]. It was administered to one male and one female member of the household above 18 years of age. Selection of the respondent for the questionnaire was done randomly from among the available eligible members of the household and who gave consent, at the time of the survey. This PHQ-9 tool has been validated for making a diagnosis of depression according to DSM-IV criteria and used in many Indian studies[27–30]. A score of 10 or above was considered as screened positive for depression [26].

Inappropriate care was a key dimension of ineffective coverage. However, we found it difficult to measure it when the providers were qualified. The household respondents were often not in a position to judge whether the treatment provided was correct. It required availability of detailed clinical data on each utilisation and clinical expertise to assess the correct treatment. It could have been feasible to ask the household respondents to give a score for quality or satisfaction but we decided against recording that as a measure of inappropriate care. Patient satisfaction is an important aspect but it is not the same as clinically correct care.

Each household respondent was also asked a direct question on whether any of the members experienced any unmet healthcare need over the preceding one year. The purpose was to compare the results of this direct question against the indirect measurement of unmet need.

### Ethics approval

Written informed consent was obtained from all respondents. The dataset was fully anonymized before analysis. The study was approved by the Institutional Ethical Committee of State Health Resource Centre (SHRC), Chhattisgarh.

### Data analysis

Cross-tabulations were applied for descriptive indicators and comparisons across socio-demographic categories. Multi-variate analyses were carried out to find out the socio-demographic categories of individuals who were likely to have greater healthcare needs. The data analysis was done in STATA 15 software.

## Results

The socio-demographic profile of the sample is given in Table 1.

### A. Perceived healthcare needs

The findings on the perceived needs were as follows (Table 1):

- 10.47% of respondents reported having experienced an acute ailment, which they perceived required healthcare in the last 15 days.
- In addition to the above, 8.18% of respondents reported having a chronic (condition persisting more than three months). This was when there was no cue or prompt. Once given a prompt with ten common chronic conditions, this went up to 10.62%.

**Table 1:** Proportion of population who had a perceived health need

	Total no in sample (n)	Self-reported acute ailment in last 15 days (%)	Self-reported Chronic Condition (%)	Self-reported Chronic Condition with prompts* (%)	Hospitalization rate - hospitalised in last 365 days (%)
**Total**	3153	10.47	8.18	10.62	5.07
**Place of residence**					
Rural	2012	10.04	6.86	8.55	4.47
Urban	1141	11.22	10.52	14.29	6.13
**Sex**					
Male	1549	8.46	8.13	10.01	3.81
Female	1604	12.41	8.23	11.22	6.30
**Age group**					
0–4	309	19.74	0.94	0.97	3.24
5–14	581	11.57	2.25	2.42	1.21
15–29	920	7.73	3.20	3.31	6.63
30–44	642	9.56	7.89	9.71	5.01
45–59	458	9.83	20.31	29.26	6.11
60 and above	243	9.88	26.75	37.04	9.05
**Social groups**					
Scheduled Tribes (ST)	667	9.71	5.85	7.43	5.65
Schedule Castes (SC)	1052	10.44	9.19	12.53	4.59
Other backward classes (OBC)	933	11.33	8.42	10.60	4.72
Others	501	9.03	13.54	18.75	5.56
**Education**					
Illiterate	1009	12.89	10.04	13.79	4.35
Primary	479	11.88	8.75	11.22	5.04
Secondary	1377	9.22	7.07	8.79	5.25
Above secondary	288	6.59	6.59	8.58	5.79
**Occupation**					
Own Agriculture	646	8.05	6.66	8.82	2.79
Daily wages worker	1621	11.29	6.42	8.57	5.74
Self-employed	407	12.53	11.30	12.29	5.16
Government employee	110	4.55	19.09	25.45	6.36
Private sector employee	319	10.66	10.97	16.30	5.96
Coal Industry Employee	50	10.00	18.18	18.18	4.00
**Wealth Index- Rural**					
Poorest	406	9.36	6.90	8.87	4.93
Poor	408	10.29	4.41	4.90	5.39
Middle	395	10.38	7.34	7.59	4.56
Rich	410	10.73	5.61	8.05	3.17
Richest	393	9.41	10.18	13.49	4.33
**Wealth Index-Urban**					
Poorest	235	15.74	7.66	10.64	5.96
Poor	220	13.18	8.18	10.00	7.27
Middle	230	5.22	10.00	13.48	6.52
Rich	226	16.81	15.93	20.35	6.64
Richest	230	5.22	10.87	16.96	4.35

The rate of perceived healthcare need/ self-reported morbidity for acute illness and for chronic illness changed with background characteristics of the place of residence (urban-rural), sex, age-group, caste, education level, occupation and income (Table 1). We expect that the more marginalized the social circumstance, the greater the morbidity, but as per this table which reports on perceived morbidity, we see that the greater the marginalization, the lesser the perceived healthcare need or reported morbidity.

The pattern of the most disadvantaged sections reporting the least healthcare needs was most accentuated in case of the chronic conditions. For instance, self-reporting of chronic ailment was 6.90% in the poorest rural quintile whereas it was 10.87% in the richest urban quintile. Gender was the only exception, with women reporting more morbidity viz. higher perceived healthcare need than men (Table 1). The above descriptive findings were confirmed in the multi-variate analysis (Additional File Table S1). The rural individuals in comparison to the urban, the scheduled tribes compared to the other social groups (castes), those in the poorest quintile compared to the better-off categories; were likely to have lower perceived health needs for chronic illness (Additional File Table S1).

### Perceived healthcare needs - that were unmet

#### Unmet need for acute ailments

Out of the total population who reported an acute ailment requiring healthcare, 12.75% did not take any treatment (Table 2). Apart from not taking treatment at all, another 27.83% took treatment from the unqualified providers. In other words, 40.58% of persons with acute ailments in the last 15 days did not receive effective health care as defined in this study (Table 3).

**Table 2:** Unmet needs out of the perceived healthcare needs

	Unmet Need for ailments in last 15 days (n = 345)		Unmet Need for chronic conditions (n = 375)	
	No treatment taken	Informal provider	No treatment taken	Informal provider
**Total**	12.75	27.83	18.40	9.07
**Residence**				
Rural	8.41	32.71	19.60	12.06
Urban	19.85	19.85	17.05	5.68
**Sex**				
Male	13.77	29.71	14.46	11.45
Female	12.08	26.57	21.53	7.18

#### Unmet need for chronic conditions

Around 27.47% of those with perceived chronic illness had unmet needs-. 18.40% took no treatment, 9.07% went to an informal provider (Table 2).

To seek care for a chronic condition patient had to travel on an average 44 km (km) for a public provider, 40 km for a qualified private provider, and 5.5 km for the informal private provider. In terms of median distance, it was 4.0 km for the public, 7.0 km for private, and 1.0 km for the informal provider. The median distance for public providers was the same (4.0 km) in rural and urban areas, whereas for the qualified private providers, the median distance a patient had to travel was 22.5 km in rural areas compared to 3.0 km in urban areas.

#### Incomplete care

On an average, patients suffering from chronic conditions discontinued their medication against healthcare professional advice for 6.06 months (or continued for 5.94 months) out of the preceding 12 months. The discontinuity was highest (10 months) in 0–4 years’ age group, where the chronic condition was a disability (cerebral palsy, autism, mental retardation, developmental delay in achieving milestone) and parents lost hope with treatment. Discontinuation of medication was relatively higher in populations belonging to lower socioeconomic status.

Out of the total patients on treatment, 23.49% got free medicines, which was marginally higher in urban areas (24.52%) than rural areas (22.50%). Availability of free medicines was higher in the poorest economic quintile (53.57%) in rural areas (Table 3).

**Table 3:** Access to free medicines for perceived health needs of chronic conditions (n = 319)

	Average duration of medicine discontinued in last 12 months against medical advice (in months)	Proportion on treatment who received free medicines (n = 305)
**Total**	6.06	23.49
**Rural-Urban divide**		
Rural	6.93	22.50
Urban	5.07	24.52
**Sex**		
Male	6.10	22.22
Female	6.03	24.56
**Age group**		
0–4	10.00	0.00
5–14	5.76	16.67
15–29	7.69	28.57
30–44	7.04	20.00
45–59	5.42	21.01
60+	5.50	28.42
**Social groups**		
Scheduled Tribe (ST)	7.54	37.88
Schedule Caste (SC)	5.85	21.88
OBC	5.71	20.42
Others/ General (GEN)	5.00	13.95
**Education**		
Illiterate	6.52	27.50
Up to primary	5.58	25.44
Up to secondary	6.66	21.52
Above secondary	5.15	14.29
**Occupation**		
Own Agriculture	6.83	20.37
Daily wages worker	6.95	18.11
Self employed	4.83	18.37
Govt. employee	5.28	25.93
Private employee	4.69	37.50
SECL Employee	4.33	60.00
**Wealth Index- Rural**		
Poorest	8.19	53.57
Poor	8.52	27.78
Middle	8.16	22.22
Rich	7.02	9.68
Richest	4.55	12.50
**Wealth Index-Urban**		
Poorest	6.14	23.53
Poor	6.06	14.81
Middle	5.02	22.58
Rich	4.50	34.78
Richest	4.50	20.59

### B. Unmet need for inpatient care

For hospitalisation or in-patient care, the survey was not able to differentiate between those needing hospitalisation and the proportion who could utilise it. The household respondents found it difficult to state their perceived need in terms of the specifying whether they needed hospitalisation or outpatient care for their illness/condition. The data thus collected on hospitalisation need was only on utilisation. It could not capture the unmet need for hospitalisation i.e., those who needed in-patient care but could not receive it.

The reported annual hospitalization rate was 5.07%, and it was higher in urban areas (6.13%) than in rural areas (4.47%). Since rural population is not expected to have lower need for inpatient care than the urban population, this difference in utilisation could reflect the forgone care.

### C. Latent healthcare needs in three tracer conditions - hypertension, diabetes, and depression

1. ***Hypertension***: Out of the total population of the age of 30 years or above, 47.42% never had blood pressure measured before in their lifetime. When screened for high blood pressure as per protocol 39.89% had high blood pressure (Table 4).

**Table 4:** Access to hypertension and diabetes screening for individuals above 30 years age and results of screening during the survey (n = 755)

	Hypertension				Diabetes			
	Blood pressure never measured before	Screening showed high blood pressure	Had been earlier diagnosed as hypertensive	Medicine being taken for hypertension	Blood glucose never measured before	Screening showed high blood sugar	Diagnosed as diabetes by a healthcare provider before	Medicine being taken for diabetes
**Total**	47.42	39.89	11.39	9.27	72.17	9.22	6.55	5.05
**Rural-Urban divide**								
Rural	59.10	37.72	6.42	5.57	73.86	6.39	4.58	3.70
Urban	28.47	43.40	19.44	15.28	69.34	13.92	9.85	7.30
**Sex**								
Male	50.00	41.67	10.71	8.93	73.39	6.79	5.50	3.98
Female	45.35	38.46	11.93	9.55	71.18	11.17	7.39	5.91
**Age group**								
30–44	51.53	27.47	3.99	2.45	77.46	5.10	1.90	1.59
45–59	46.07	44.74	14.61	13.11	69.38	12.20	9.30	7.36
60+	41.36	56.79	20.99	16.67	66.25	12.58	11.25	8.13
**Social groups**								
Scheduled Tribe (ST)	59.92	38.17	6.20	4.96	80.83	5.91	3.33	2.92
Schedule Caste (SC)	42.57	38.00	13.86	10.89	71.58	10.87	8.42	6.32
Other backward caste (OBC)	44.19	39.94	12.79	10.17	65.97	10.75	7.76	5.97
Others/ General (GEN)	26.47	48.53	19.12	17.65	73.02	11.11	9.52	6.35
**Education**								
Illiterate	59.31	42.79	10.82	8.66	75.88	10.22	7.46	6.14
Up to primary	50.40	40.73	11.69	10.08	76.76	9.62	7.05	5.39
Up to secondary	40.00	33.51	9.19	7.57	68.72	8.99	5.59	3.91
Above secondary	24.18	43.33	16.48	12.09	56.47	5.88	4.71	3.53
**Occupation**								
Own Agriculture	69.72	42.96	8.45	7.04	75.00	3.60	3.57	2.14
Daily wages labour	49.88	36.79	8.64	6.67	79.54	8.76	5.63	4.86
Self employed	39.77	40.00	9.09	6.82	66.67	12.20	8.33	5.95
Government Employee	16.00	52.00	36.00	24.00	54.17	8.33	4.17	4.17
Retired govt. employee	17.65	64.71	17.65	17.65	23.53	29.41	29.41	17.65
Private sector employee	15.87	39.68	22.22	22.22	51.61	12.90	9.68	6.45
Coal Industry Employee	33.33	46.67	33.33	26.67	53.33	20.00	13.33	13.33
**Wealth Index- Rural**								
Poorest	73.04	42.61	5.22	4.35	84.21	3.57	0.88	0.88
Poor	78.35	31.25	3.09	2.06	93.75	4.21	2.08	0.00
Middle	56.12	32.65	5.10	4.08	68.75	4.26	3.13	2.08
Rich	56.52	42.03	4.35	4.35	60.29	8.82	7.35	7.35
Richest	25.00	40.70	14.77	13.64	54.12	12.94	11.76	9.41
**Wealth Index-Urban**								
Poorest	46.55	44.83	15.52	15.52	89.09	9.09	9.09	5.45
Poor	27.87	27.87	13.11	6.56	70.49	13.11	3.28	3.28
Middle	34.55	49.09	14.55	12.73	65.38	21.15	15.38	13.46
Rich	25.45	43.64	27.27	18.18	52.73	12.73	9.09	5.45
Richest	8.47	52.54	27.12	23.73	68.63	14.00	13.73	9.80

2. ***Diabetes***: Similarly, in the 30 years or above age group in our sample 72.17% never had blood glucose measured before in their lifetime (Table 4). When screened during the survey, 9.22% of them had high blood sugar (Table 4). Disaggregating by background characteristics, this unmet need was usually higher for the socioeconomically disadvantaged groups. For example, the chances of being screened before for blood glucose was 3.66 times higher (95% CI: 1.71–7.81) in the richest quintile compared to the poorest quintile (Additional File Table S2).
  Known diabetics and hypertensive i.e., the self-reported or perceived healthcare needs, were greater in higher socioeconomic groups (Table 4).
3. ***Depression***: As revealed by the screening conducted during the survey, the prevalence of depression in our sample (PHQ-9 score > = 10) was 11.9% in the adult (18 years plus) age-group. Of those who screened positive less than 1 per cent were on normative treatment despite over 80% having significant difficulties in activities of daily living (**Additional File Table S3**). In effect almost all cases of depression were unmet needs for healthcare.

### D. Findings from the direct question on unmet healthcare needs

Individuals were also asked a direct question about whether any of their perceived healthcare needs, had remained unmet in the last 365 days, and 9.61% of respondents reported this positive and it was significantly higher in upper socioeconomic compared to lower socioeconomic population group (Additional File Table S4).

### D. summarizing unmet healthcare needs

There were various kinds of unmet healthcare needs which were presented in the three subsections A to C. These findings are summarized and synthesized in Table 5.

**Table 5:** Matrix of unmet healthcare needs

	Unmet Healthcare Needs	
	When health care needs were perceived as such	When healthcare needs were unperceived
***Usually recognised as unmet needs***:	Treatment not received at all: Examples ¬ 12.75% of people suffering from acute ailments in the last 15 days did not take treatment at all. ¬ 18.40% of people having chronic conditions did not take treatment	Latent diseases: Examples ¬ In terms of preventive healthcare needs, 47.42% of individual (30 years or above age) never had their blood pressure measured before in their lifetime. Similarly, 72.17% never had their blood glucose measured before. ¬ 95% of those identified with likelihood of depression had not sought any healthcare to get the diagnosis or treatment as they did not know they could be suffering from depression.
***Measurable but often ignored as an unmet need***:	Treatment taken from an unqualified informal provider: ¬ 27.83% of people suffering from acute ailments in the last 15 days took treatment from the informal provider. ¬ 9.06% of people having a chronic condition in the last 365 days took treatment from the informal provider.	Incomplete care: ¬ Patients suffering from chronic conditions discontinued medicine for an average of 6.06 months out of the past 12 months.
***Difficult to measure and often ignored***:	Inappropriate care by formal provider and the patient could report inappropriate care when asked	Inappropriate care by formal provider but the patient was unable to assess whether it was appropriate or not

## Discussion

Our study shows a variety of unmet healthcare needs. The indicators of physical availability of services or insurance coverage were not able to reflect these access problems, all of which happened in a context where there was physical access to healthcare providers and an insurance cover. This reflects the difference between measuring ‘access’ and ‘effective access’ [16].

Most studies tend to report unmet needs as the self -reported morbidity for which no healthcare was received. Household surveys that collect information on self-reported morbidity, services utilization, and out-of-pocket expenditures are the primary data sources for measuring access. In Indian context, the health rounds of National Sample Survey (NSS) are a key source. However, this study shows a range of unmet needs that such surveys will fail to pick up. Studies on access like the health surveys of NSS use response to a single question – “have you any illness now or in last 15 days?” to record morbidity and, therefore healthcare needs [4]. The latest round of NSS (2017-18) reported 4.9% of the Chhattisgarh’s population with any illness in past 15 days. In comparison, our survey reported a much greater rate of 10.47% experiencing an ailment in past 15 days.

The NSS does not report the prevalence of chronic diseases as it does not include a separate question for chronic diseases. Other surveys like LASI and SAGE follow a single question with a list of leading questions asking specifically whether for the presence of the more common chronic illness [24]. The single question under-estimates chronic illness. In this study with a single question, the morbidity rate is 8.18% and with the prompts, the prevalence rate of chronic illness increases to 10.62%. Studies have shown that special interviewing techniques –respondents instruction, feedback, long question, probing – used singly or in combination increases reporting rates of chronic conditions [31].

But either way, surveys like the NSS miss latent illnesses. As shown in this study, a major part of unmet healthcare needs takes this form. Indian guidelines prescribe measurement of blood pressure once a year for each individual above age of thirty but nearly half the population in this age had never undergone screening for hypertension. When screened in our survey, 39.89% of them were found with high blood pressure. Even if a smaller proportion of them get confirmed as hypertensive, it would lead to a big jump over the self-reported burden of chronic diseases. A similar increase is expected if the diabetes burden gets fully uncovered. The more recent surveys are adding blood pressure and blood sugar to the survey, but this only uncovers latent illness in only a few of the NCDs [32]. Similar latency could extend to many more NCDs, mental illness and even chronic communicable diseases like tuberculosis, HIV/AIDS and leprosy.

Incomplete care was another form of unmet health needs. Most surveys miss this dimension. We found that almost every patient of a chronic disease missed medication for a part of the year and the average duration without medication was of 6 months in a year. This is too huge a gap for the care to be effective. Only a quarter of the chronic patients could access free medicines.

The qualified private providers were available within short distances for urban patients but not in rural areas. For rural patients, the public providers were available within a short distance. Overall, the unqualified providers scored over the formal providers - public and private in terms of proximity. The study showed that using distance or physical accessibility as a main measure of access can have limitations. Having a provider available nearby did not ensure that the various kinds of health needs were met.

Categorizing the care from informal providers as ‘inappropriate care’ is relatively a grey area. Not all researchers categorize care from informal providers as unmet health care needs. Many have made a case for acknowledging this as a legitimate market response to healthcare needs [33]. However, our discussion is in the context of measuring progress to UHC. In the road-maps to UHC in India, access to unqualified providers has no place hitherto and, therefore, would not qualify as effective access to healthcare. Our study showed that a big part of outpatient care was being handled by them. Concerns have been raised about the quality of care they provide [34, 35].

Merely being treated by a healthcare provider is not effective access. The care provided should conform to a normative understanding of what is the required healthcare. There could be excessive, inessential care, which is a problem in its own right, but if appropriate care is not part of the care provided, it remains an unmet healthcare need. The Tanahashi framework refers to inappropriate care as that is not effective, and so does the UHC definition [16]. Previous studies done in India have cautioned about inappropriate care [36, 37]. Unmet healthcare needs in terms of the inappropriateness of care can be perceived and non-perceived. Our study was not able to measure it quantitatively. To measure inappropriate care as a researcher is a challenging dimension, especially in a context where health records are very incomplete or absent, and communication to patients have been scanty. Patients were often not in a position to assess whether they received the correct care. Inappropriate care can conceal unmet health needs. Only grossly inappropriate care as communicated by a physician or as uncovered by a researcher in consultation with a physician could be categorized as such. The increase in inappropriate care has been reported from other studies in India [38]and elsewhere [39]. Considering the importance of this dimension, there is a need to keep looking for ways to measure it, including assessments using qualitative methods.

Past studies, mostly done in high income countries, measure unmet healthcare needs via asking one-liner questions:“ have you had unmet healthcare needs in the last 365 days?”. This study finds that such a question seriously under-estimates even the perceived healthcare needs. In our study, 40.58% of individuals in acute ailment and 27.47% in the chronic ailments either did not had treatment at all or received from an unqualified provider, not to speak about incomplete and inappropriate care. Our study found that the disadvantaged population was likely to under-perceive it further. Earlier studies too have reported this and attributed it to characteristics of the patient and the limitations of a one-year recall. [40, 41].

A higher level of unmet healthcare needs in the lower socioeconomic population measures unmet healthcare needs essential to understand the inequity in access to care in society. This study notes that perceived health care needs (all) are higher in upper socioeconomic and less marginalized groups. If we include latent healthcare needs – the higher incidence of (perceived) healthcare needs in higher economic quintiles (mainly due to chronic illness and higher hospitalizations) reduces significantly. Amartya Sen had explained this phenomenon of India states with higher socioeconomic states showing greater morbidity in morbidity surveys than lower socioeconomic states in terms of “positional objectivity’ [42]. Thus, Kerala, which has the highest literacy and density of health care facilities, and relatively high income per capita, has the highest morbidity among all Indian states in self-reporting based on NSS surveys [4]. On the other hand, poorer states with lower literacy and educational attainment and more poverty are more at risk for disease but paradoxically report lesser morbidity than Kerala [4]. The Global Burden of Disease Study, 2017,shows that the poorer states have a higher disease burden. For instance, diseases burden, which was measured in terms of age-specific, disability adjusted life year (DALY) was significantly higher in Chhattisgarh (1.71) than Kerala (1.00), but self-reporting diseases burden was significantly higher in Kerala than Chhattisgarh [43, 44].

The causes of the high proportion of unmet needs are evidenced from survey data. One major problem is that patients often access public healthcare facilities only to be told that the required services for that specific disease condition are not available. This is a consequence of health policies that have promoted very selective healthcare packages for government-provided primary healthcare- not only in the primary health centre but also in the district hospitals [45, 46]. Thus, there is either no population-based effort at uncovering latent needs or promoting appropriate healthcare-seeking behaviour for many healthcare needs. For many others where the need is perceived, though there is an accessible healthcare facility, the appropriate care is not available, and the patient is referred away. Recognizing this and as an effort to address this problem, universal coverage with government-funded public health insurance is introduced, but in the absence of private providers who can provide appropriate care, it does not lead to increased access. Rather because there are a reimbursement provisions available, inappropriate care increases in the local private sector. So often, the desired level of care is available in the government medical college hospital of the distant city and appropriate private care is also available at the same distance. The simple learning is that improving mechanisms of financing health care and risk pooling is quite inadequate where the problems are largely of access. In the area studied, the immediate requirement was to strengthen the public health system and provide comprehensive primary health care. It is worth noting that the government has initiated a program called the Health and Wellness Centres for this. However, while early reports are encouraging, it is still too early to say whether it will address the considerable problem of unmet needs and equity in access to effective essential health care.

Our major learning from this study is that uncovering unmet healthcare needs is a key task for equitable design of health care systems and measuring progress towards UHC. Since the prevalence of unmet healthcare needs is more in the poorer and more marginalized sections, measuring differentials in unmet healthcare needs across sub-groups with lower social and economic status is essential.

The ways in which the household surveys were tweaked in the current study allowed them to measure many important types of unmet needs. This shows the potential household surveys hold for better measurement of unmet needs and thus helping in assessing progress on UHC. Findings of this study also provides insights about how to improve standard Indian household survey to better capture unmet healthcare needs as follows: First, the single question under-estimates chronic illness. Even with perceived needs, using a set of questions instead of only one probe significantly increases the reporting for chronic conditions. Second, most of the national level surveys either miss or under represent the informal healthcare provider. Many times they categorize informal healthcare provider and formal healthcare provider together. Our study shows a novel methodology of identifying the informal healthcare provider, which is very important for the measurement of unmet healthcare needs. Third, this study shows the importance of capturing the incomplete care which is missing in national level surveys in India. Capturing the incomplete care will help in capturing the access to healthcare, unmet healthcare needs, and allocating resources in the country. Finally, inappropriate care is an important area to capture which requires incorporation of qualitative exploration along with self-rating of care by patient itself. More research will be needed to cover all dimensions of unmet needs comprehensively and this may require developing the quantitative tools further and to complement them with qualitative methods.

This study has its limitations. The morbidity prevalence rates measured in this study should be interpreted with caution because of several limitations. First, the effect of seasonality is not captured since it’s a one-time cross-section study. Second, measurement of the latent needs are limited to three tracer conditions- if more had been included, more latent needs were likely to be uncovered. Finally, findings of the study only refer to district studies, and it is not automatically representative for India as a whole. However, this survey provides important insights about unmet healthcare needs which is missing in national level surveys in India. Also, findings of this study can be used as a baseline to measure progress over time in the study area which is also warranted in other districts or regions of India.

## Conclusions

Measurement of healthcare needs and measurement of unmet (or unfulfilled) healthcare needs are multidimensional and complex. Yet they are essential to understand the gaps in effective access to healthcare and plan to close these gaps to monitor progress towards universal healthcare. Further, because unmet needs, in contrast to perceived needs, are much higher in population sub-groups of lower socioeconomic status and greater marginalization, ensuring equity in access requires such measurement. Indicators measuring progress towards UHC should go beyond indicators reflecting insurance enrolment and utilization of services and include a few select indicators reflecting unmet needs and further seek to measure them across different relevant population sub-groups. Only then progress towards UHC measured and correct planning be done. Suitably designed household surveys offer a significant potential to uncover unmet healthcare needs.

## Electronic supplementary material

Below is the link to the electronic supplementary material.

Supplementary Material 1

## Data Availability

The datasets used and/or analysed during the current study are available from the corresponding author and State Health Resource Centre, Chhattisgarh on reasonable request.

## List of Abbreviations

BADL: Basic activities of daily living
IADL: Instrumental activities of daily living
LASI: Longitudinal ageing study in India
MSBY: Mukhyamantri Swasthya Bima Yojana
NFHS: National Family Health Survey
PHQ-9: Patient health questionnaire-9
PPS: Probability proportional to size
RSBY: Rashtriya Swasthya Bima Yojana
SAGE: Study on global AGEing
UHC: Universal Health Coverage
UMPCE: Usual monthly per capita expenditure
