# Supplementary material for: Measurement of unmet healthcare needs to assess progress on universal health coverage - exploring a novel approach based on household surveys
Source: BMC Health Serv Res. 2023 May 23;23:525. doi: 10.1186/s12913-023-09542-0 (PMC10207793; doi:10.1186/s12913-023-09542-0)
Supplement: Supplementary file 1 — Supplementary Material 1 [file 12913_2023_9542_MOESM1_ESM.docx]

**Additional File Table-S1: Results of logistic regression models for factors affecting perceived healthcare needs (n=3153)**

|  | Ailment in last 15 days | | Hospitalization | | Chronic condition | |
| --- | --- | --- | --- | --- | --- | --- |
|  | Odds ratio | p-value | Odds ratio | p-value | Odds ratio | p-value |
| **Rural-Urban divide** |  |  |  |  |  |  |
| Rural | 1.00 |  | 1.00 |  | 1.00 |  |
| Urban | 1.23 (0.90-1.66) | 0.18 | 1.61 (1.06-2.45) | 0.02 | 1.32 (0.95-1.83) | 0.08 |
| **Sex** |  |  |  |  |  |  |
| Male | 1.00 |  | 1.00 |  | 1.00 |  |
| Female | 1.56 (1.23-1.99) | <0.01 | 1.88 (1.33-2.67) | <0.01 | 1.18 (0.90-1.55) | 0.20 |
| **Age group** |  |  |  |  |  |  |
| 0-4 | 1.00 |  | 1.00 |  | 1.00 |  |
| 5-14 | 0.45 (0.29-0.70) | <0.01 | 0.27 (0.09-0.76) | 0.01 | 2.29 (0.63-8.24) | 0.20 |
| 15-29 | 0.33 (0.20-0.53) | <0.01 | 1.73 (0.77-3.90) | 0.18 | 4.56 (1.32-15.74) | 0.01 |
| 30-44 | 0.41 (0.26-0.65) | <0.01 | 1.33 (0.59-2.99) | 0.48 | 11.71 (3.52-38.96) | <0.01 |
| 45-59 | 0.42 (0.26-0.67) | <0.01 | 1.87 (0.85-4.12) | 0.11 | 43.10 (13.27-140.01) | <0.01 |
| 60+ | 0.45 (0.27-0.77) | <0.01 | 3.26 (1.46-7.27) | <0.01 | 61.37 (18.77-200.56) | <0.01 |
| **Social groups** |  |  |  |  |  |  |
| Scheduled Tribe (ST) | 1.00 |  | 1.00 |  | 1.00 |  |
| Schedule Caste (SC) | 1.05 (0.72-1.52) | 0.80 | 0.71 (0.41-1.20) | 0.20 | 1.60 (1.06-2.42) | 0.02 |
| Other backward caste (OBC) | 1.08 (0.81-1.44) | 0.59 | 0.70 (0.47-1.05) | 0.09 | 1.44 (1.02-2.01) | 0.03 |
| Others/ General (GEN) | 0.80 (0.48-1.36) | 0.43 | 0.65 (0.33-1.26) | 0.21 | 1.92 (1.17-3.15) | <0.01 |
| **Education** |  |  |  |  |  |  |
| Illiterate | 1.00 |  | 1.00 |  | 1.00 |  |
| Up to primary | 1.19 (0.85-1.68) | 0.30 | 1.75 (1.04-2.94) | 0.03 | 1.01 (0.71-1.44) | 0.93 |
| Up to secondary | 1.10 (0.72-1.67) | 0.65 | 1.52 (0.82-2.81) | 0.18 | 0.83 (0.54-1.28) | 0.42 |
| Above secondary | 0.89 (0.52-1.51) | 0.66 | 1.54 (0.76-3.13) | 0.23 | 0.42 (0.25-0.73) | <0.01 |
| **Occupation** |  |  |  |  |  |  |
| Own Agriculture | 1.00 |  | 1.00 |  | 1.00 |  |
| Daily wages labourer | 1.42 (1.01-2.00) | 0.04 | 2.11 (1.23-3.61) | 0.01 | 0.99 (0.68-1.43) | 0.95 |
| Self employed | 1.80 (1.15-2.81) | 0.01 | 1.99 (1.00-3.99) | 0.05 | 0.96 (0.60-1.55) | 0.89 |
| Government Employee | 0.68 (0.25-1.79) | 0.44 | 2.84 (1.14-7.08) | 0.03 | 2.45 (1.31-4.57) | <0.01 |
| Private sector salaried employee | 1.42 (0.85-2.37) | 0.17 | 2.32 (1.11-4.83) | 0.02 | 1.36 (0.82-2.26) | 0.23 |
| Coal Industry Employee | 1.24 (0.45-3.41) | 0.67 | 1.27 (0.27-5.91) | 0.76 | 1.36 (0.54-3.34) | 0.50 |
| **Wealth Index** |  |  |  |  |  |  |
| Poorest | 1.00 |  | 1.00 |  | 1.00 |  |
| Poor | 1.23 (0.84-1.78) | 0.28 | 1.22 (0.71-2.09) | 0.45 | 0.93 (0.58-1.49) | 0.78 |
| Middle | 1.16 (0.79-1.72) | 0.44 | 1.04 (0.59-1.82) | 0.88 | 1.18 (0.75-1.85) | 0.47 |
| Rich | 1.10 (0.72-1.70) | 0.63 | 1.00 (0.54-1.84) | 0.98 | 1.58 (0.99-2.52) | 0.05 |
| Richest | 0.90 (0.54-1.48) | 0.67 | 0.64 (0.31-1.31) | 0.23 | 2.08 (1.25-3.46) | <0.01 |
| **Insurance coverage** |  |  |  |  |  |  |
| No insurance | 1.00 |  | 1.00 |  | 1.00 |  |
| Enrolled in publicly funded health insurance | 1.04 (0.79-1.37) | 0.763 | 0.77 (0.53-1.14) | 1.19 | 1.14 (0.81-1.59) | 0.42 |
| Constant | 0.11(0.06-0.18) | <0.01 | 0.01 (0.004-0.02) | <0.01 | 0.003 (0.001-0.013) | <0.01 |

**Additional File Table S2: Chances of having screened before for blood pressure (hypertension) and blood glucose (diabetes) in the community for age 30 years or above - results of logistic regression models**

|  | Hypertension (n=755) | | Diabetes (n=733) | |
| --- | --- | --- | --- | --- |
|  | Odds ration | p-value | Odds ration | p-value |
| **Rural-Urban divide** |  |  |  |  |
| Rural | 1.00 |  | 100 |  |
| Urban | 1.51 (0.98-2.32) | 0.06 | 0.51 (0.32-0.80) | <0.01 |
| **Sex** |  |  |  |  |
| Male | 1.00 |  | 1.00 |  |
| Female | 1.35 (0.94-1.94) | 0.09 | 1.18 (0.80-174) | 0.40 |
| **Age group** |  |  |  |  |
| 0-4 | NA |  |  |  |
| 5-14 | NA |  |  |  |
| 15-29 | NA |  |  |  |
| 30-44 | 1.00 |  | 1.00 |  |
| 45-59 | 1.59 (1.06-2.38) | 0.02 | 1.67 (1.07-2.60) | 0.02 |
| 60+ | 2.6 (1.59 – 4.28) | <0.01 | 2.22 (1.32-3.74) | 0.03 |
| **Social groups** |  |  |  |  |
| Scheduled Tribe (ST) | 1.00 |  | 1.00 |  |
| Schedule Caste (SC) | 0.96 (0.55-1.68) | 0.91 | 1.25 (0.68-2.31) | 0.45 |
| Other backward caste (OBC) | 0.95 (0.63-1.42) | 0.80 | 1.83 (1.16-2.89) | 0.01 |
| Others/ General (GEN) | 0.68 (0.32-1.45) | 0.32 | 0.75 (0.33-1.68) | 0.49 |
| **Education** |  |  |  |  |
| Illiterate | 1.00 |  | 1.00 |  |
| Up to primary | 1.48 (0.96-2.28) | 0.07 | 0.88 (0.54-1.43) | 0.61 |
| Up to secondary | 2.25 (1.33-3.81) | <0.01 | 1.44 (0.82-2.54) | 0.20 |
| Above secondary | 2.50 (1.22-5.11) | 0.01 | 1.74 (0.85-3.57) | 0.13 |
| **Occupation** |  |  |  |  |
| Own Agriculture | 1.00 |  | 1.00 |  |
| Daily wages labourer | 2.07 (1.32-3.25) | <0.01 | 0.86 (0.52-1.42) | 0.57 |
| Self employed | 1.45 (0.77-2.71) | 0.24 | 1.25 (0.64-2.44) | 0.51 |
| Government Employee | 4.27 (1.62-11.23) | <0.01 | 3.85 (1.69-8.77) | <0.01 |
| Private Sector Salaried employee | 4.25 (1.84-9.80) | <0.01 | 2.32 (1.11-4.84) | 0.02 |
| Coal Industry Employee | 1.62 (0.48-5.39) | 0.43 | 2.54 (0.79-8.23) | 0.12 |
| **Wealth Index** |  |  |  |  |
| Poorest | 1.00 |  | 1.00 |  |
| Poor | 2.41 (1.45-4.02) | <0.01 | 2.13 (1.13-4.02) | 0.02 |
| Middle | 2.43 (1.40-4.20) | <0.01 | 2.85 (1.48-5.46) | <0.01 |
| Rich | 4.30 (2.37-7.78) | <0.01 | 4.27 (2.18-8.35) | <0.01 |
| Richest | 6.28 (3.09-12.77) | <0.01 | 3.66 (1.71-7.81) | <0.01 |
| **Insurance coverage** |  |  |  |  |
| **No insurance** | 1.00 |  | 1.00 |  |
| RSBY/MSBY | 0.80 (0.49-1.32) | 0.40 | 0.69 (0.39-1.21) | 0.20 |

**Additional File Table S3: Proportion of population screened positive for depression and difficulty in their day today life based on their response to PHQ-9 score, in >=18-year age group (n=839)**

|  | PHQ-9 score >= to 10 (n=839) | Difficulty in the day-to-day life if PHQ>=10 (n=81) |
| --- | --- | --- |
| **Total** | 11.20 | 86.17 |
| **Rural-Urban divide** |  |  |
| Rural | 12.33 | 89.06 |
| Urban | 9.38 | 80.00 |
| **Sex** |  |  |
| Male | 11.20 | 85.37 |
| Female | 11.21 | 86.79 |
| **Age group** |  |  |
| 18-29 | 6.28 | 69.23 |
| 30-44 | 10.06 | 84.38 |
| 45-59 | 11.48 | 91.67 |
| 60+ | 23.81 | 92.00 |
| **Social groups** |  |  |
| Scheduled Tribe (ST) | 11.70 | 93.55 |
| Schedule Caste (SC) | 15.38 | 85.00 |
| Other backward caste (OBC) | 10.48 | 79.49 |
| Others/ General (GEN) | 5.56 | 100.00 |
| **Education** |  |  |
| Illiterate | 16.95 | 90.00 |
| Up to primary | 11.57 | 89.29 |
| Up to secondary | 9.77 | 88.46 |
| Above secondary | 6.49 | 60.00 |
| **Occupation** |  |  |
| Own Agriculture | 12.88 | 95.24 |
| Labourer/ Work on daily wages | 11.01 | 83.67 |
| Self employed | 13.59 | 85.71 |
| Government Employee | 21.74 | 100.0 |
| Retired Government Employee | 27.27 | 33.33 |
| Private Sector Salaried employee | 2.47 | 100.00 |
| Coal Industry Employee | 0.00 | - |
| **Wealth Index- Rural** |  |  |
| Poorest | 11.72 | 100.00 |
| Poor | 13.89 | 100.00 |
| Middle | 12.62 | 76.92 |
| Rich | 10.71 | 77.78 |
| Richest | 12.50 | 83.33 |
| **Wealth Index-Urban** |  |  |
| Poorest | 15.00 | 100.00 |
| Poor | 12.31 | 62.50 |
| Middle | 8.47 | 60.00 |
| Rich | 1.61 | 100.00 |
| Richest | 7.41 | 75.00 |

**Additional File Table S4: Factors affecting perception of having an unmet healthcare need in past one year (direct question)- results of logistic regression model**

|  | Unmet health needs from people’s perception | |
| --- | --- | --- |
|  | Odds ration | p-value |
| **Rural-Urban divide** |  |  |
| Rural | 1.00 |  |
| Urban | 2.04 (1.46-2.85) | <0.01 |
| **Sex** |  |  |
| Male | 1.00 |  |
| Female | 1.42 (1.09-1.84) | 0.01 |
| **Age group** |  |  |
| 0-4 | 1.00 |  |
| 5-14 | 0.52 (0.26-1.01) | 0.05 |
| 15-29 | 1.15 (0.62-2.13) | 0.65 |
| 30-44 | 2.30 (1.31-4.04) | <0.01 |
| 45-59 | 2.51 (1.44-4.37) | <0.01 |
| 60+ | 5.90 (3.39-10.26) | <0.01 |
| **Social groups** |  |  |
| Schedule Tribe (ST) | 1.00 |  |
| Schedule Caste (SC) | 1.38 (0.93-2.03) | 0.10 |
| Other backward caste (OBC) | 1.01 (0.74-1.39) | 0.92 |
| Others/ General (GEN) | 1.07 (0.64-1.79) | 0.79 |
| **Education** |  |  |
| Illiterate | 1.00 |  |
| Up to primary | 0.90 (0.64-1.27) | 0.57 |
| Up to secondary | 0.71 (0.46-1.09) | 0.12 |
| Above secondary | 0.49 (0.28-0.85) | 0.01 |
| **Occupation** |  |  |
| Own Agriculture | 1.00 |  |
| Daily wages labourer | 1.46 (1.02-2.11) | 0.04 |
| Self employed | 1.50 (0.92-2.45) | 0.10 |
| Government Employee | 2.27 (1.17-4.41) | 0.02 |
| Private Sector Salaried employee | 1.08 (0.60-1.93) | 0.79 |
| Coal Industry Employee | 0.77 (0.22-2.71) | 0.68 |
| **Wealth Index** |  |  |
| Poorest | 1.00 |  |
| Poor | 0.76 (0.52-1.11) | 0.16 |
| Middle | 0.47 (0.30-0.72) | <0.01 |
| Rich | 0.47 (0.30-0.74) | <0.01 |
| Richest | 0.50 (0.30-0.83) | 0.01 |
| **Insurance coverage** |  |  |
| **No insurance** | 1.00 |  |
| RSBY/MSBY | 1.05 (0.77-1.42) | 0.74 |
| Constant | 0.05 (0.02-0.09) | <0.01 |
